# Fibre phantom generation using *FibreSimulator*: an open-source Python tool

**DOI:** 10.1107/S1600577526001918

**Published:** 2026-03-25

**Authors:** Mary Chris Roperos Go, Daniël M. Pelt, Anirudh Kohli, Philip J. Withers, K. Joost Batenburg

**Affiliations:** a Leiden Institute of Advanced Computer Science Universiteit Leiden Leiden The Netherlands; b Henry Royce Institute, Department of Materials University of Manchester Manchester United Kingdom; Paul Scherrer Institute, Switzerland; EPFL, Switzerland

**Keywords:** radiography, computational modelling, computed tomography, *FibreSimulator*

## Abstract

Fibre-reinforced polymer composites are utilized across many industries for their stiffness and strength. Visualization of their internal structure is critical to understand their mechanical properties, with computed tomography serving as a popular non-destructive method. While classical tomographic reconstruction algorithms do not rely on training data, modern machine-learning-based methods require large datasets that realistically reflect experimental imaging conditions. However, acquiring such datasets for composites is challenging due to limited access to the ground truth and the high cost of repeated scans. There is therefore a need for realistic, controllable and labelled synthetic phantoms. To address this gap, we present *FibreSimulator*, an open-source Python tool that generates 3D synthetic phantoms of unidirectional fibre-reinforced polymers with customizable material properties, fibre orientations and geometrical features. The simulator is integrated with the *ASTRA* toolbox to simulate tomographic scans of the generated phantoms. We demonstrate its capacity through experiments evaluating how tomographic scan settings affect reconstruction and segmentation. Simulated tomographic experiments show that reducing the number of X-ray projections or lowering beam intensity (*i.e.* increasing noise) leads to blurred fibre boundaries, overestimated fibre diameters and fewer detectable fibres. While these qualitative effects are well known, *FibreSimulator* enables their systematic and quantitative investigation under fully controlled conditions, with access to the ground truth. In particular, by varying acquisition parameters and composite properties, we observe a nonlinear trend in detection accuracy arising from undersampling and fibre-overlap artefacts. Such controlled studies are difficult to perform experimentally due to cost, limited repeatability and absence of the ground truth. These results highlight *FibreSimulator* as a valuable tool for optimizing parameters and guiding experimental design under controllable conditions.

## Abstract 2

*FibreSimulator* is an open-source tool for generating realistic 3D phantoms of unidirectional fibre-reinforced polymers, enabling customizable fibre structures and simulating computed tomography scanning for algorithm development and validation.

## 1. Introduction

Fibre-reinforced polymer (FRP) composites are widely researched due to their extensive application across various industries, including energy production systems (Ennis *et al.*, 2023 ▸). These materials are valued for their specific stiffness, strength and design flexibility (Alzahrani *et al.*, 2025 ▸). The mechanical performance of FRPs is inherently linked to their internal structure, where fibre orientation, distribution and matrix bonding play crucial roles. Therefore, a comprehensive understanding of the composite’s internal structure is essential for optimizing material properties and ensuring structural reliability. However, visualization poses challenges for inspection and analysis due to the micrometre-scale fibre diameters. To obtain a complete 3D representation of the internal fibre arrangement, advanced imaging techniques are required.

X-ray computed tomography (CT) has become a key tool for this purpose (Garcea *et al.*, 2018 ▸; Salling *et al.*, 2022 ▸), as it provides non-destructive, high-resolution 3D imaging of FRP materials. In contrast to conventional destructive methods, such as the microtomy and grinding and polishing method (Abdelkader *et al.*, 2022 ▸), which physically sections the specimen, CT enables volumetric analysis without altering the sample. By producing detailed 3D representations of FRPs through CT, this method allows researchers to see the distribution and orientation of fibres, as well as defects within the polymer matrix. The 3D representations provide a more comprehensive view of how fibres are embedded within the matrix, how stress is transferred between phases (Almeida *et al.*, 2025 ▸), and how porosity (Wang *et al.*, 2025 ▸), delamination (Yu *et al.*, 2016 ▸) and fibre misalignment (Wang *et al.*, 2021 ▸) affect mechanical behaviour. The tomographic imaging pipeline typically consists of data acquisition, reconstruction, denoising, segmentation and analysis, with methods at each stage advancing rapidly (Withers *et al.*, 2021 ▸).

Recently, machine-learning algorithms (Greffier *et al.*, 2022 ▸; Alagic *et al.*, 2022 ▸; Yıldızcan *et al.*, 2024 ▸; Bellens *et al.*, 2024 ▸) have gained popularity alongside traditional image reconstruction and processing techniques. These data-driven approaches excel at learning complex patterns and structures from large datasets, allowing them to generalize within the distribution on which they were trained. They have significantly advanced the state of the art in image quality enhancement through methods such as denoising (Burger *et al.*, 2012 ▸; Zhang *et al.*, 2017 ▸), deblurring (Pan *et al.*, 2016 ▸) and limited-angle reconstruction (Barutcu *et al.*, 2021 ▸). Their end-to-end optimization makes them particularly attractive for fibre-imaging pipelines, including fibre segmentation (Badran *et al.*, 2022 ▸; Badran *et al.*, 2020 ▸; Guo *et al.*, 2023 ▸), as well as defect identification (Bang *et al.*, 2020 ▸).

Despite the growing interest in deep-learning-based tomographic reconstruction, post-processing and quantitative analysis, a major challenge remains: the limited availability of quality datasets tailored to tomographic algorithm development. Unlike large-scale computer vision datasets such as CIFAR (Krizhevsky, 2012 ▸), ImageNet (Deng *et al.*, 2009 ▸) and MNIST (Deng, 2012 ▸), CT datasets are often scarce, proprietary or highly specialized. Many of these datasets originate from synchrotron facilities or industrial applications and are often restricted to private use, making them difficult to access for broader scientific use. Moreover, acquiring high-quality CT data is expensive and time-consuming, and requires careful control of scanning conditions and preprocessing. To address these challenges, using synthetic phantoms can support the development of data-driven algorithms for tomographic data. The generation of such phantoms is motivated by the following considerations:

(i) To enable *fair and reproducible* comparison between algorithms by providing datasets with a known ground truth.

(ii) To support the *training and validation of data-driven models*, which require large and diverse datasets often unavailable from real-world sources.

(iii) To allow *controlled variation of object complexity*, enabling a systematic comparison of algorithm performance across different levels of difficulty.

To properly support the development and evaluation of data-driven algorithms for tomographic data, datasets should ideally satisfy the following requirements (Pelt *et al.*, 2022 ▸):

(i) The datasets should be *challenging*, making accurate reconstruction non-trivial, revealing algorithmic strengths and weaknesses.

(ii) The datasets should be *representative* of real-world samples, typical noise levels, imaging artefacts and acquisition setups.

(iii) The datasets should be *flexible*, allowing controlled variation in parameters.

(iv) The datasets should support *data-driven* methods, providing sufficient diversity and volume for training and testing of machine-learning models.

A phantom generator that meets these requirements would enable fair and reproducible evaluation of traditional and data-driven algorithms for tomographic data.

For some application areas, the lack of ground truth CT datasets is solved within the tomographic community by building phantom generators for testing and comparing tomographic reconstruction methods, *e.g.* foam phantoms (Pelt *et al.*, 2022 ▸) and earlier work on generating multiple sets of experimental phantom data (Sitek *et al.*, 2006 ▸). These phantom generators create synthetic volumetric data that can be used to evaluate imaging techniques under controlled conditions, allowing researchers to systematically analyse the effects of noise, resolution and algorithmic parameters on reconstruction quality. Although these phantom generators have proven useful for general tomographic research, they are not specifically tailored for FRPs. Instead, several domain-specific software tools have been developed to simulate FRP structures, *TexGen* (Long & Brown, 2011 ▸), *WiseTex* (Lomov, 2021 ▸) and *Fibersim* (Siemens, 2024 ▸). *TexGen* and *WiseTex* are well established tools for generating idealized texting and tow-based geometries, offering a user-friendly graphical interface. However, a previous study (Nemeth *et al.*, 2010 ▸) has reported limitations in consistently generating well conditioned finite element meshes for both tows and matrix materials, particularly when moving beyond idealized configurations. Moreover, these tools are primarily designed for geometric and mechanical modelling rather than for producing voxel-based ground truth volumes suitable for CT simulation. On the other hand, *Fibersim* is a commercial composite design platform primarily intended for industrial laminate design and manufacturing workflows. While it offers advanced CAD-based modelling capabilities, *Fibersim* is proprietary and closed-source. The constraints make it less suitable for users to customize or extend them by adding new defect models and non-standard geometrical configurations. As a result, the generated datasets are limited in their ability to reflect a diverse range of fibre characteristics.

To address the limitations of existing FRP simulation tools, we present a Python-based fibre simulator. It aims to generate phantom fibres that mimic real-world fibre structures found in unidirectional FRPs with customizable features. This flexibility provides a controlled environment where researchers can generate desired fibre volumes with realistic variations and geometrical features. Additionally, the simulator enables users to simulate scanning the volume and vary the scanning parameters through the *ASTRA* toolbox (van Aarle *et al.*, 2015 ▸). This feature allows the assessment of different scanning configurations before conducting an actual CT scan. It also plays a crucial role in providing ground truth data for testing and evaluating reconstruction strategies, which are essential for improving accuracy in real-world tomographic imaging. Furthermore, the simulator is written in Python, benefiting from its extensive library support and large community, enabling easy extension for custom fibre behaviour and geometrical features. This simulator is the initial version, with plans to add additional features such as more complex geometrical features, varied fibre orientations and advanced scanning configurations.

This paper is structured as follows. In Section 2, we outline the detailed steps involved in generating single fibres and fibre phantom volumes, along with different types of geometrical features and a comprehensive discussion of the parameters of the phantom volume. In Section 3, we demonstrate various case studies that show the capabilities of the simulator to mimic real-world data and explore the effects of varying scanning configurations, such as noise levels and the number of projections taken over the full 180° angular range, and the impact of different fibre characteristics, such as the number of fibres. In Section 4, we summarize the key contributions of this paper.

## 2. Methods

In real-world X-ray CT scans of fibre-reinforced composites, fibres appear as elongated cylinder structures embedded within a resin matrix. These scans often reveal complex features such as fibre misalignment, kinks, diameter variations, and occasional voids or air pockets.

To facilitate the development of fibre phantom volumes, we simulate such structures as voxel volumes. Each voxel in our generated volume is assigned a discrete intensity value that represents a material class. This voxel-based representation provides a controlled and customizable way to reproduce the visual and structural complexities present in real CT datasets. Furthermore, tomographic simulation frameworks such as *ASTRA* operate natively on voxel grids. The voxel-based approach enables efficient handling of fibre overlap and intersections, and allows defects such as voids, notches or local material variations to be introduced through simple volumetric operations. The proposed framework prioritizes computational efficiency, ease of modification and tight integration with CT simulation, while maintaining sufficient flexibility for future extensions.

**Figure 1:**
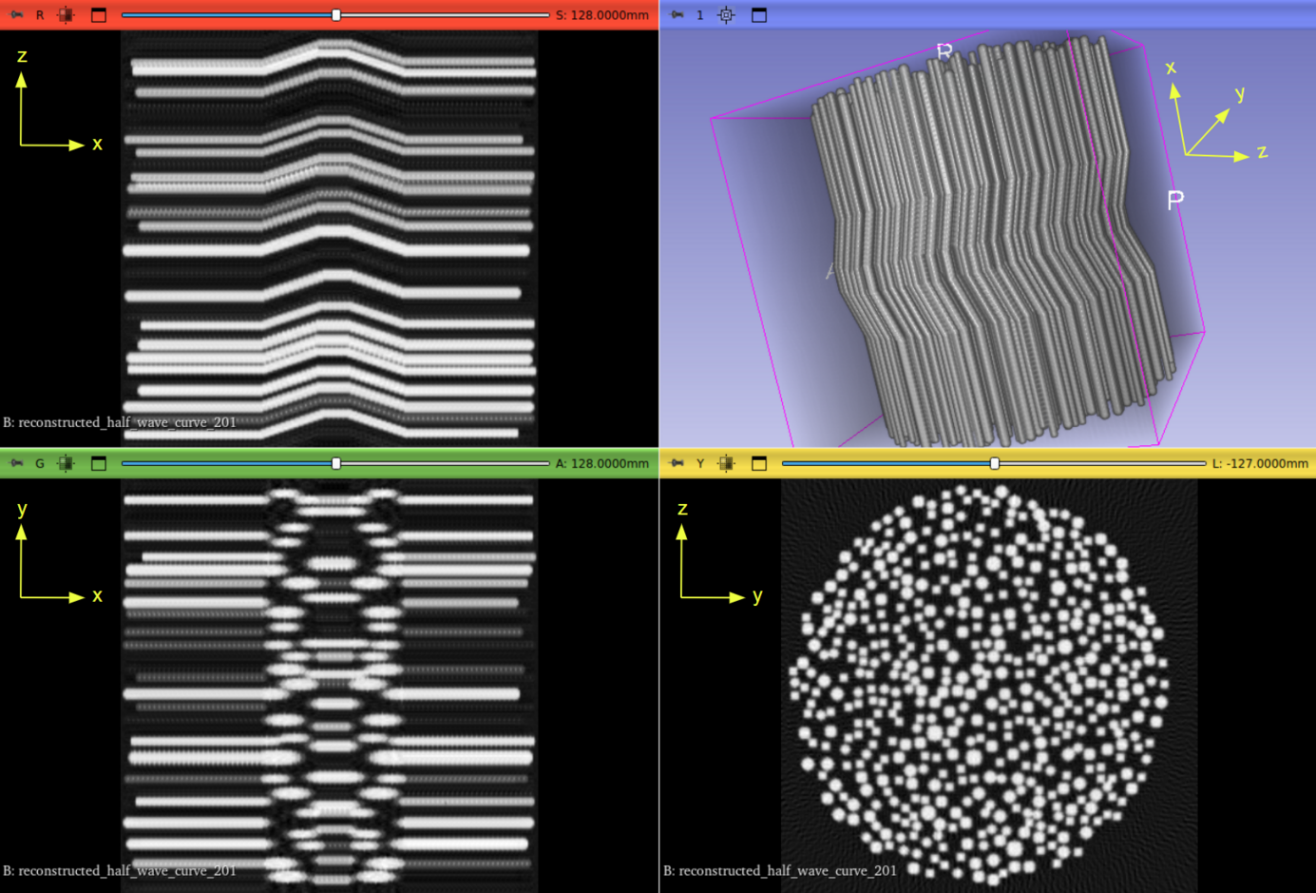
Visualization in the *3D Slicer* software (Kikinis *et al.*, 2014 ▸) of a synthetic fibre volume. In this example, a half-wave curve phantom volume is shown. The top left panel (red border) shows the sagittal view. The top right panel presents a 3D rendering of the full fibre volume. The bottom left panel (green border) corresponds to the coronal view, slicing the structure from front to back. The bottom right panel (yellow border) displays the axial view, a cross section from top to bottom, highlighting the circular distribution of fibres.

Fig. 1 ▸ illustrates an example of a generated synthetic volume, viewed in *3D Slicer* (Kikinis *et al.*, 2014 ▸).

This section outlines the methodology for generating synthetic fibre phantoms. We first define the mathematical concepts, then describe the construction of individual fibres and the full-volume generation process. We also present a visual example and describe additional features such as fibre orientation and voids.

### 2.1. Notations and concepts

We define a fibre phantom as a set of *N*_*f*_ fibres: 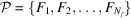. Each fibre *F*_*m*_ is modelled as a sequence of *N*_*s*_ points 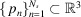, where each point *p*_*n*_ = (*x*_*n*_, *y*_*n*_, *z*_*n*_) denotes the centre of a sphere that composes a fibre, and all spheres share a common radius *r*. Here *N*_*s*_ denotes the number of points describing a fibre and may vary between fibres, depending on the starting position and the imposed minimum and maximum axial bounds of the simulation volume.

Thus, a single fibre can be written as 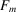 = 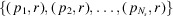. Here, *F*_*m*_ refers to the mathematical representation of the fibre, which is a sequence of centre points and a fixed radius. It does not yet describe the actual region in space that the fibre occupies. That region is denoted by
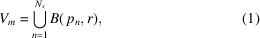
where 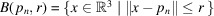 denotes a solid sphere of radius *r* centred at point *p*_*n*_. Therefore, each fibre is a continuous 3D volume formed by connecting overlapping spheres. The total volume occupied by all fibres in the phantom is then
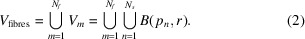
This continuous volume 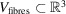 serves as the geometric model of the fibre phantom before discretization.

#### 2.1.1. Fibre growth rule

**Figure 2:**
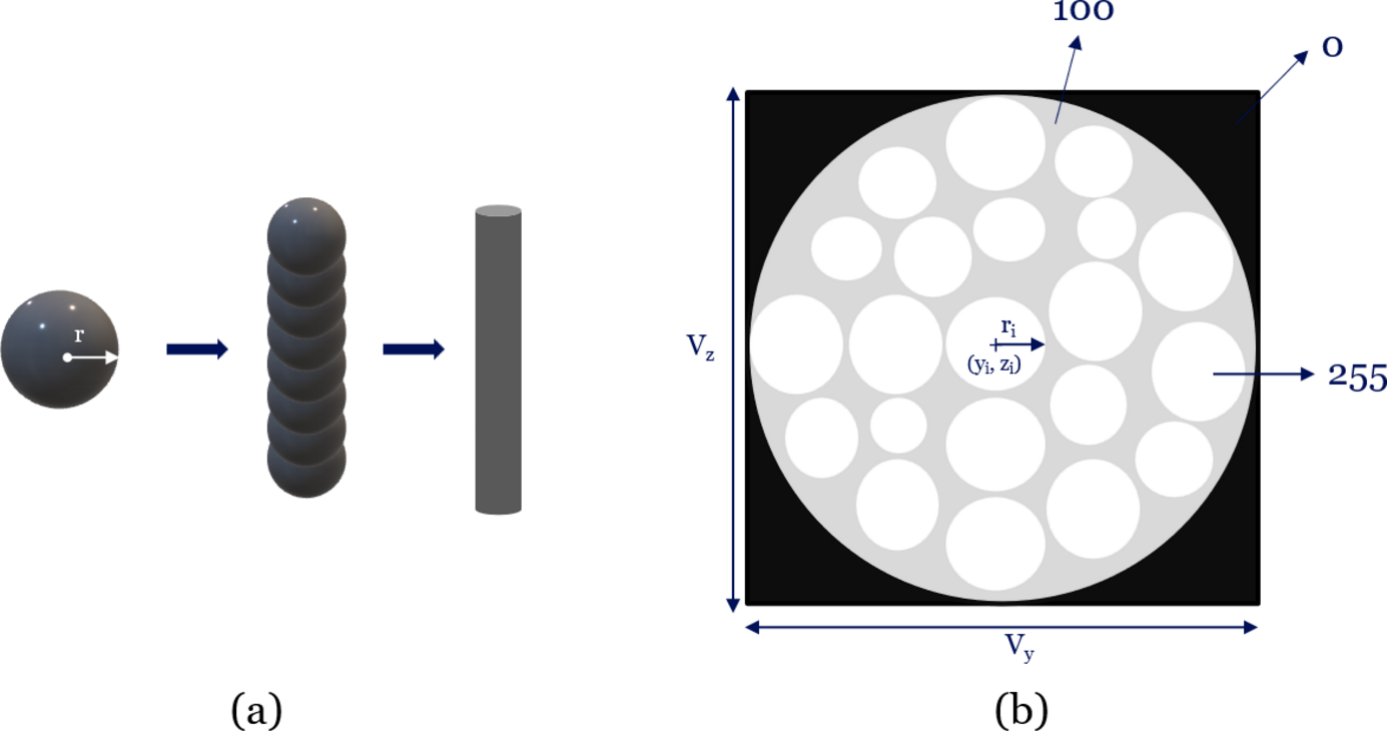
A schematic representation of (*a*) the composition of a fibre and (*b*) the axial slice of a fibre phantom.

Fibres are generated as sequences of spheres in continuous 3D space, as seen in Fig. 2 ▸(*a*). The spheres are positioned closely together, approximating a continuous cylinder. This representation follows a well established modelling approach for filament-like objects, as previously described in the ball–chain model of Altendorf & Jeulin (2011 ▸). Fig. 2 ▸(*b*) shows a schematic representation of an axial slice of a fibre phantom, where (*V*_*x*_, *V*_*y*_, *V*_*z*_) denote the number of voxels in each dimension. Each fibre starts from an initial point *p*_1_, which is randomly sampled within a valid region. Specifically, the point must lie inside the *pipe*, which constrains where fibres can grow. The pipe is modelled as a cylindrical region defined along the *x* axis. For any position *x*, the centre of the pipe cross section in the *y*–*z* plane is fixed and centred at (center_*y*_, center_*z*_), and the pipe has a constant radius *r*_pipe_. Since the pipe is cylindrical and extends infinitely along the *x* axis, points are constrained only in the *y*–*z* cross sections.

To simplify the generation process and avoid complex behaviours such as curling or looping, we assume that all fibres grow strictly along the *x* axis. This restriction is intentionally adopted as a starting point that reflects common unidirectional fibre-reinforced composite configurations. While more complex fibre architectures can be represented by existing simulation tools, the present work focuses on establishing a well defined and reproducible baseline geometry from which the influence of scanning conditions can be studied systematically.

In practice, an initial point sampled at integer coordinates (*i*, *j*, *k*) is considered to be a valid starting point if its centre at real coordinates (*x*, *y*, *z*) = (*i*, *j*, *k*) satisfies the condition 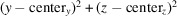

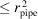
. Once a valid starting point is found, the fibre begins at a point *p*_1_ = (*i*, *j*, *k*) in real coordinates, and subsequent points are computed iteratively using an update rule *U* in continuous space: *p*_*n*+1_ = *U*(*p*_*n*_).

Before a new point *p*_*n*+1_ = (*x*_*n*+1_, *y*_*n*+1_, *z*_*n*+1_) is added, a virtual sphere of radius *r* is placed at that point, and it is accepted only if:

(i) All nearby grid points within the sphere lie inside the pipe.

(ii) None of the points is already labelled as part of another fibre.

A point (*i*, *j*, *k*) is considered inside the sphere if its centre coordinates satisfy (*i* − *x*_*n*+1_)^2^ + (*j* − *y*_*n*+1_)^2^ + (*k* − *z*_*n*+1_)^2^ ≤ *r*^2^. Note that the sphere constraint requires all three spatial dimensions to be checked, unlike the pipe constraint, which only considers the *y*–*z* cross section since the pipe extends infinitely along the *x* axis. In our framework, we assume the orientation of the fibres is along the *x* axis. Therefore, we define an update rule *U*^+^ that adds a step in the positive *x* direction during fibre growth. If the newly generated point is invalid, the algorithm may attempt to grow in the reverse direction using a different update rule *U*^−^, which adds a step in the negative *x* direction. If both directions fail and the minimum length has not yet been achieved, the process restarts with a new starting point.

Furthermore, the present implementation constrained fibres to grow predominantly along the *x* axis, with deviations restricted to the *y*–*z* plane. This assumption reflects alignment found in many unidirectional FRPs and allows controlled simulation of curvature and waviness while avoiding intersections and looping behaviour. However, this restriction limits the representation of more general fibre architectures observed in practice, such as global misalignment, out-of-plane waviness, local twisting or fully 3D bending induced by manufacturing defects.

Although fibres are generated with a preferred direction in this work, the growth mechanism is formulated in continuous 3D space and decoupled from the voxelization and CT simulation stages. This mechanism allows increased flexibility in fibre orientation to be incorporated at the growth stage while preserving the existing pipeline.

#### 2.1.2. Voxel representation

Once all the fibres are generated in continuous space, they are converted onto the voxel grid with dimensions 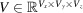, where each voxel holds a scalar intensity value representing the average CT attenuation coefficient of the material present in that voxel. A voxel is marked as a fibre if its centre lies inside any sphere that defines a fibre. Such voxels are set to *c*_*f*_. Voxels outside the pipe are assigned the air value *c*_*a*_, while voxels in the pipe that do not contain fibres are filled with the resin value *c*_*r*_. This process results in a discrete phantom volume that mimics real-world composite materials, with clearly labelled regions for fibres, resin and air.

### 2.2. Fibre phantom volume generation

We generate the phantom fibre by fibre, starting from *m* = 1 up to *m* = *N*_*f*_. For each fibre *F*_*m*_, where *m* = 1,…, *N*_*f*_, the algorithm proceeds as follows (see also Appendix *A*[App appa]):

(1) Initialize a starting point. Randomly sample a point *p*_1_ = 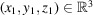 such that:

 (*a*) It lies within the pipe, defined by: (*y*_1_ − center_*y*_)^2^ + (*z*_1_ − center_*z*_)^2^ ≤ 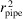.

 (*b*) A sphere of radius *r* centred at *p*_1_ does not overlap with existing fibre voxels. This rule checks by simulating the sphere at the voxel level, verifying for each voxel at the grid position (*i*, *j*, *k*) around *p*_1_ that (*i* − *x*_1_)^2^ + (*j* − *y*_1_)^2^ + (*k* − *z*_1_)^2^ ≤ *r*^2^, and that the voxel is not already labelled as fibre.

(2) Grow the fibre. Starting from *p*_1_, a fibre path is constructed iteratively using a user-defined update rule: *p*_*n*+1_ = *U*(*p*_*n*_). The same validity checks mentioned in step (1) are performed on the new point. If *p*_*n*+1_ is invalid, the algorithm attempts to grow in the opposite direction using a reverse update rule *U*^−^. If both directions fail and the fibre has not yet reached the minimum length, the current fibre is discarded, and a new *p*_1_ is sampled.

(3) Store the fibre. Once a valid fibre of *N*_*s*_ points has been generated, it is added to the global fibre set.

(4) Repeat. Steps (1)–(3) are repeated until the total number of fibres *N*_*f*_ is reached.

(5) Discretized and label. After all fibres have been generated in continuous space, each point is expanded into a solid sphere using the same Euclidean condition. Voxels within each sphere are labelled with the fibre intensity value *c*_*f*_. Remaining voxels inside the pipe are filled with the resin value *c*_*r*_, while voxels outside the pipe are set to the air value *c*_*a*_.

In general, fibres are generated sequentially using a rejection-based placement strategy that prevents overlap between fibres by discarding candidate points that intersect previously placed fibres. This approach guarantees non-overlapping fibre volumes but does not explicitly model physical compaction or packing dynamics. As a result, local fibre density may differ from packing patterns observed in real-life FRPs.

At higher requested fibre volume fractions, geometric constraints increasingly limit the available space for additional fibres, and the generator may fail to place further fibres within a finite number of attempts. No post-placement relaxation or repulsion steps are applied to redistribute fibres once placed. The placement strategy prioritized control over fibre count rather than physically accurate packing statistics.

### 2.3. Fibre phantom volume example

A fibre volume refers to the 3D structure of a fibre-reinforced material. In real composite materials, fibre volume exhibits a wide range of structural variations, including straight or curved (kinking) fibres, differences in fibre diameters, and the presence of manufacturing defects such as voids and holes.

**Figure 3:**
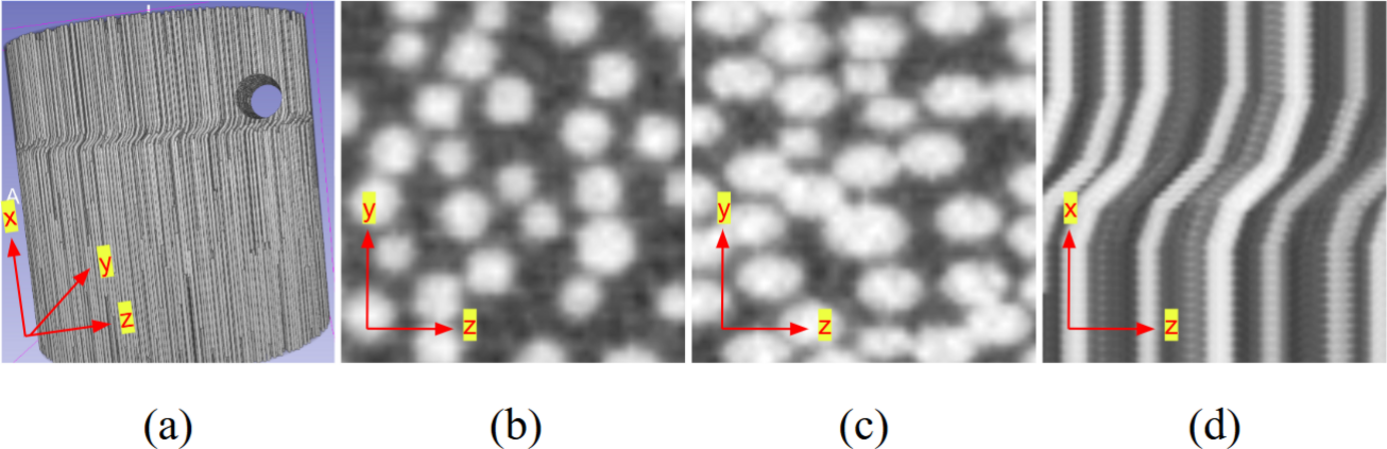
Visualization of a reconstructed phantom volume containing a kink and a hole: (*a*) 3D view of a fibre phantom volume, (*b*) magnified view of the axial slice, (*c*) magnified view of the axial slice at the kink and (*d*) magnified view of the sagittal slice. The set of parameters used to generate the volume is shown in Table 3 (in the supporting information).

These structural variations and defects, commonly observed in real-world CT data, can also be simulated using the simulator. Fig. 3 ▸ shows an example of a generated [800 × 800 × 800] reconstructed fibre phantom volume, illustrating the simulator’s ability to reproduce features such as kinking, varying diameters and voids. These examples highlight the structural complexity that the simulator can achieve. A 3D representation is shown in Fig. 3 ▸(*a*). Furthermore, Fig. 3 ▸(*b*) shows the varying fibre radii, Fig. 3 ▸(*c*) shows the axial slice at a kink, exhibiting the cross section of a slanted fibre, and Fig. 3 ▸(*d*) displays the sagittal view that highlights the fibre structure at a kink.

### 2.4. Other features

Features such as fibre orientation, embedded defects and voids are commonly observed in manufactured composites and play a critical role in determining their mechanical performance. Understanding and simulating them are essential for closely replicating real-world composite volumes.

These features often arise during the manufacturing process and are known to significantly influence the strength and reliability of composite structures. As FRPs are increasingly used in various applications, manufacturing-induced defects have been more extensively documented and analysed in the literature (Zhang *et al.*, 2019 ▸; Wanhill *et al.*, 2015 ▸; Cai, 2020 ▸). Consequently, a thorough investigation of these structural irregularities remains essential to ensure the development of robust materials.

To account for this, we incorporated the simulation of these features, aiming to generate more realistic phantoms that reflect imperfections found in actual composite materials.

#### 2.4.1. Fibre orientation

Stacking consecutive points to make a single fibre offers flexibility in determining the fibre’s geometry. In this work, fibre orientation refers to the way the fibre changes direction as it grows, determined by the sequential placement of points. At each step, the simulator computes a direction vector **d**_*n*_, which determines where the next point will be placed. The fibre grows incrementally using a simple update rule:

(i) Forward growth: *U*^+^(*p*_*n*_) = *p*_*n*_ + **d**_*n*_ · *r*.

(ii) Backward growth: *U*^−^(*p*_*n*_) = *p*_*n*_ − **d**_*n*_ · *r*.

Here, **d**_*n*_ is a direction vector and *r* is the fibre radius. In this implementation, the radius serves two roles: it defines the thickness of the fibre (as each sphere has a radius *r*), and it also determines the distance between consecutive midpoints. The growth direction **d**_*n*_ is recalculated at every step and depends on the type of fibre being simulated. Currently, the simulator supports five orientation modes, each with its own rule for computing **d**_*n*_:

(i) Straight fibres: direction is constant **d**_*n*_ = (1, 0, 0). The fibre grows straight along the *x* axis with no deviation. Random perturbations may be added in the *y* and *z* directions for slight jaggedness.

(ii) Kink curve fibres: direction changes from straight to bent and back, based on the *x* position. The angle θ_*n*_ varies from 0° to 45° near a central kink point and then returns: **d**_*n*_ = 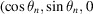).

(iii) C-curve fibres: the fibre gradually bends in the *y* direction. The direction vector includes a *y* component that increases linearly with distance from a defined bend centre *t*_*c*_: **d**_*n*_ = [1, (*x*_*n*_ − *t*_*c*_)/*r*_*c*_, 0], where *r*_*c*_ controls the sharpness of the bend.

(iv) Full-wave curve fibres: the fibre oscillates following a full sinusoidal wave. The direction angle θ_*n*_ changes sinusoidally across the *x* range: **d**_*n*_ = 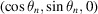 and θ_*n*_ = 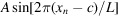, where *A* is the amplitude, *L* is the wavelength and *c* is the wave centre.

(v) Half-wave curve fibres: similar to the full wave, but covers only one oscillation: **d**_*n*_ = 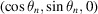 and θ_*n*_ = 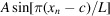.

This step-by-step update approach provides flexibility while maintaining precise control over fibre geometry.

#### 2.4.2. Geometrical features

**Figure 4:**
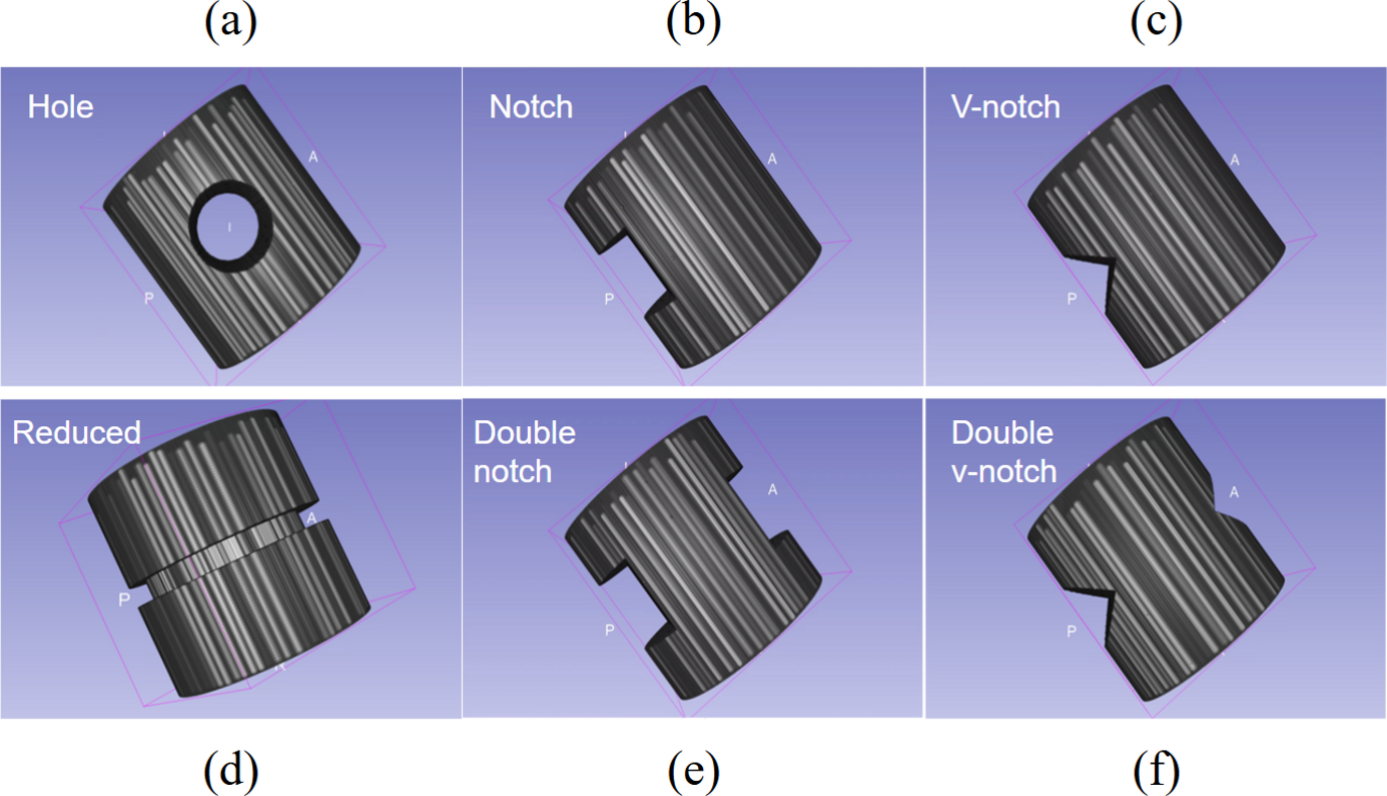
Variants of defect geometries modelled in FRPs: holes, notches, V-notches and reduced-diameter section.

The current version of the simulator incorporates multiple types of geometrical features (see Fig. 4 ▸), which are commonly encountered by researchers. These types of features were prioritized for modelling due to the availability of real-world data, enabling qualitative comparison. These features are generated by modifying an already initialized simulated full volume. Specific regions of the volume are removed to create the desired defect shape. For instance, a circular region is subtracted from the volume to form a hole. Similarly, square and V-shaped regions are carved out from the fibre volume at predetermined locations for notch features. Starting with a complete volume and introducing these features through targeted removal allows for control over the defect’s shape and size.

#### 2.4.3. Voids

In a real-world fibre composite, voids can be present within the resin of fibre composites due to manufacturing imperfections (Mehdikhani *et al.*, 2019 ▸). In the simulator, these voids are represented as small spheres embedded within the fibre volume. Following the same approach used for geometrical features, the voids are created by starting with a complete volume and assigning resin voxels (*c*_*r*_) to be air voxels (*c*_*a*_). Each void is generated as a sphere with a fixed radius of 1 voxel (*r*_void_ = 1) and, by default, the simulator creates 50 voids (*N*_voids_ = 50). These small spheres are randomly positioned within the volume to mimic voids that naturally occur during fibre manufacturing. While not exposed as a user parameter, the values for *N*_voids_ and *r*_void_ can be modified directly in the source code.

### 2.5. Volume and scanning parameters

The simulator provides users with control over the generation of fibre volume by allowing customization of various parameters. By exploring a wide range of fibre configurations and tomographic setups, the simulator is a versatile tool for generating fibre volumes. Table 1 ▸ and Table 2 ▸ outline the parameters for volume generation and virtual scanning configurations.

#### 2.5.1. Scope of CT simulation physics

The simulated projection data are generated using a simplified X-ray attenuation model based on Beer–Lambert law, as provided by the *ASTRA* toolbox. While this model captures the dominant effects of absorption and photon statistics, it does not explicitly include additional physical phenomena commonly observed in real micro-CT systems such as beam hardening, scattering, detector blur and non-linear detector response. The impact of excluding such additional effects on downstream task accuracy can depend on the specific task and application domain (Andriiashen *et al.*, 2024 ▸).

The simulator is therefore intended primarily for algorithmic benchmarking and methodological development rather than instrument-accurate replication of specific CT systems. This design choice prioritizes reproducibility, interpretability and the availability of the ground truth, allowing controlled evaluation of reconstruction, segmentation and learning-based methods under well defined conditions.

## 3. Case studies

The purpose of these experiments is to evaluate the performance and capabilities of the simulator by qualitatively comparing the generated phantoms with real-world fibre samples and analysing the impact of varying volume and tomographic reconstruction parameters.

A key structural parameter throughout the following experiments is the fibre volume fraction (FVF), defined as the proportion of the total volume occupied by fibres: 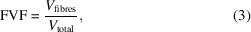
where *V*_fibres_ denotes the total volume occupied by fibres and *V*_total_ is the volume of the full phantom. It is a quantitative measurement that serves as a critical parameter in characterizing the structural composition of the fibre volume. A higher FVF indicates a denser fibre network, which often correlates with increased mechanical strength. Conversely, a lower FVF fraction suggests a more porous or loosely packed structure. In the context of phantom generation, FVF can be systematically varied by manipulating two key parameters: the number of fibres and the radius of individual fibres.

Accordingly, we explore three types of case studies:

(i) Reconstruction examples. This case study highlights the simulator’s ability to generate realistic fibre structures by varying FVF through changing the number of fibres and adjusting the radius of individual fibres. We also show how simulated experimental CT conditions, such as projection count, angular range and noise, affect reconstruction quality. An example of a phantom volume is then compared with real-world X-ray CT scans to illustrate visual similarities.

(ii) Varying tomographic imaging conditions while keeping fibre structure fixed. Here, we assess how different tomographic parameters affect reconstruction quality when the underlying fibre phantom is constant. We vary the number of projections and the initial X-ray intensity, which simulates the dose, and reconstruct the volumes. To quantify the effects, we apply imaging processing techniques to detect fibres and compute their average diameter and count. These metrics are compared with the ground truth phantom to determine which scanning conditions yield the most accurate results.

(iii) Varying fibre characteristics while keeping imaging conditions fixed. In this final case study, we explore how different levels of FVF affect fibre detectability under a fixed, limited-angle scanning setup. The aim here is to understand how dense configurations affect the accuracy of fibre count estimation. The main metric is the error rate between the detected and true number of fibres. These experiments help identify a range of FVF at which fibres are most distinguishable and errors are minimized.

The code is publicly available on GitHub (Go *et al.*, 2024 ▸) and is compatible with Windows and Linux operating systems. Generated phantoms and their corresponding projections are stored in the assigned folder as NIfTI (Cox *et al.*, 2004 ▸) files. Additionally, the metadata for the parameters used in the simulation are saved in an HDF5 (Koranne, 2011 ▸) file to ensure reproducibility.

The repository is organized in a modular manner to facilitate reuse and extension. The main entry point is provided in main.py, while all simulation and scanning parameters are defined in the user-editable parameters.json file. Core functionality is implemented in the fiber_phantom folder, which contains modules for fibre growth, defect modelling and volume construction. This structure allows users to easily modify fibre behaviour, add defect types and vary the scanning configurations. It provides a clear starting point for extending the simulator. Furthermore, the repository provides installation instructions, a description of each file and sample photos of the generated phantoms.

The experiments detailed in this paper were conducted on a workstation equipped with an AMD Ryzen 7 7800X3D 8-core processor and an NVIDIA GeForce RTX 4080 GPU, both operating under Ubuntu 22.04.04 LTS.

### 3.1. Reconstruction examples

**Figure 5:**
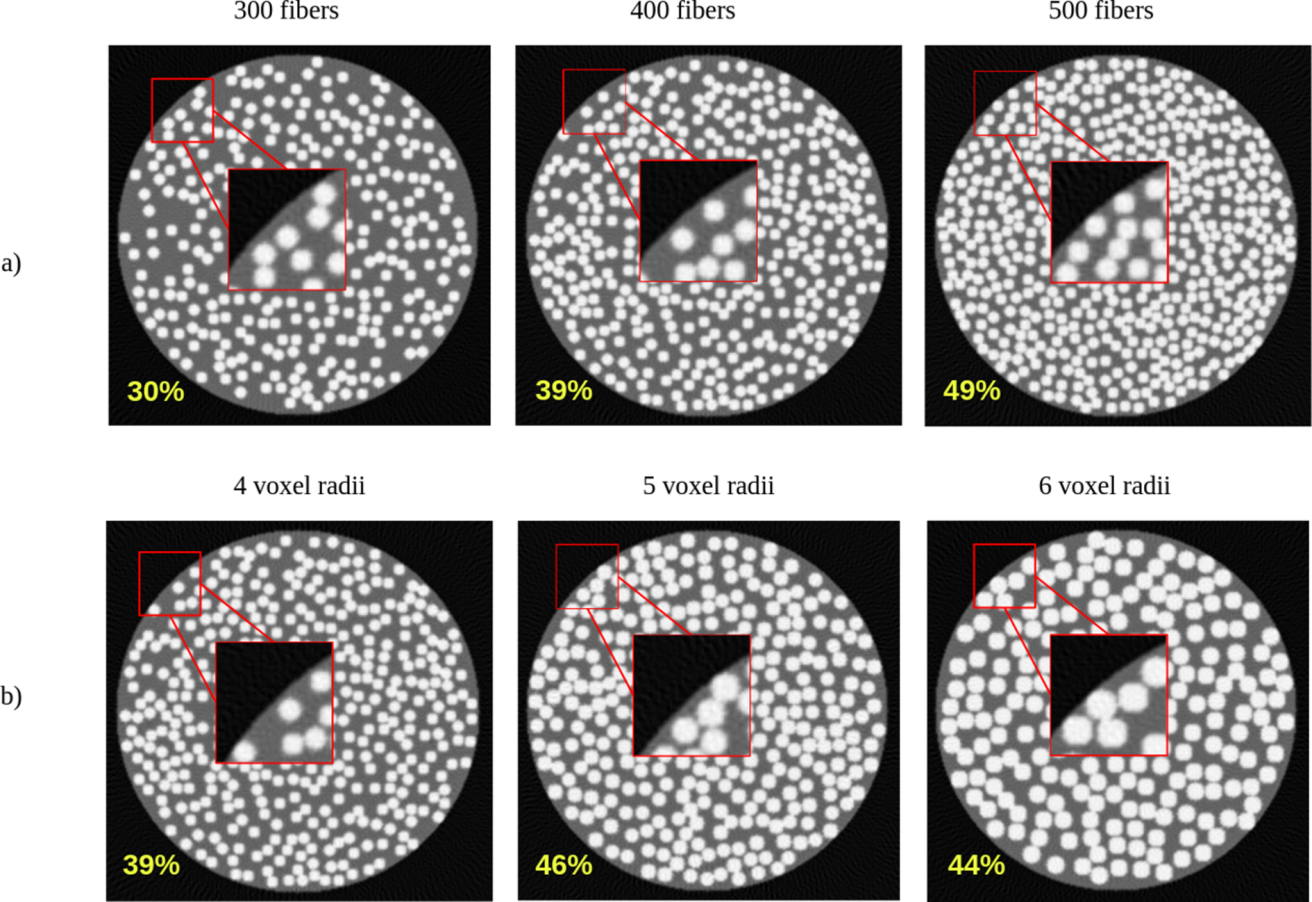
Middle axial slices of [256 × 256 × 256] generated phantoms: (*a*) with increasing number of fibres, and (*b*) with increasing voxel radius of the individual fibres. The corresponding FVF for each case is also indicated. The zoomed regions were selected from the same corner location in each slice to provide a consistent local comparison of fibre packing density and diameter variations across configurations. The parameters used to generate these volumes are given in Table 3 (in the supporting information).

To evaluate the capabilities of the simulator, we present several examples of generated phantoms that demonstrate how the FVF can be varied in two distinct ways. The first approach to modifying the FVF involves adjusting the number of individual fibres within a fixed volume. By increasing the number of fibres, as illustrated in Fig. 5 ▸(*a*), the simulator achieves a denser configuration, resulting in a higher FVF. This method maintains a constant fibre diameter while altering the overall occupancy of the space, allowing for controlled variations in density without changing the physical characteristics of each fibre.

The second method involves altering the voxel radius of the fibres, as shown in Fig. 5 ▸(*b*). By increasing the radius of each fibre, the fibres occupy more space within the volume, leading to a higher FVF. Conversely, decreasing the radius results in a lower FVF, enabling simulations of more loosely packed fibrous structures. This approach is particularly useful when modelling systems where the fibre thickness plays a significant role in material behaviour or imaging contrast. As expected, increasing the voxel radius not only increases the volume occupied by each fibre but also reduces the total number of fibres that can fit within the same volume due to spatial constraints. Together, these two methods provide flexible control over the FVF, allowing the simulator to replicate different fibre configurations.

**Figure 6:**
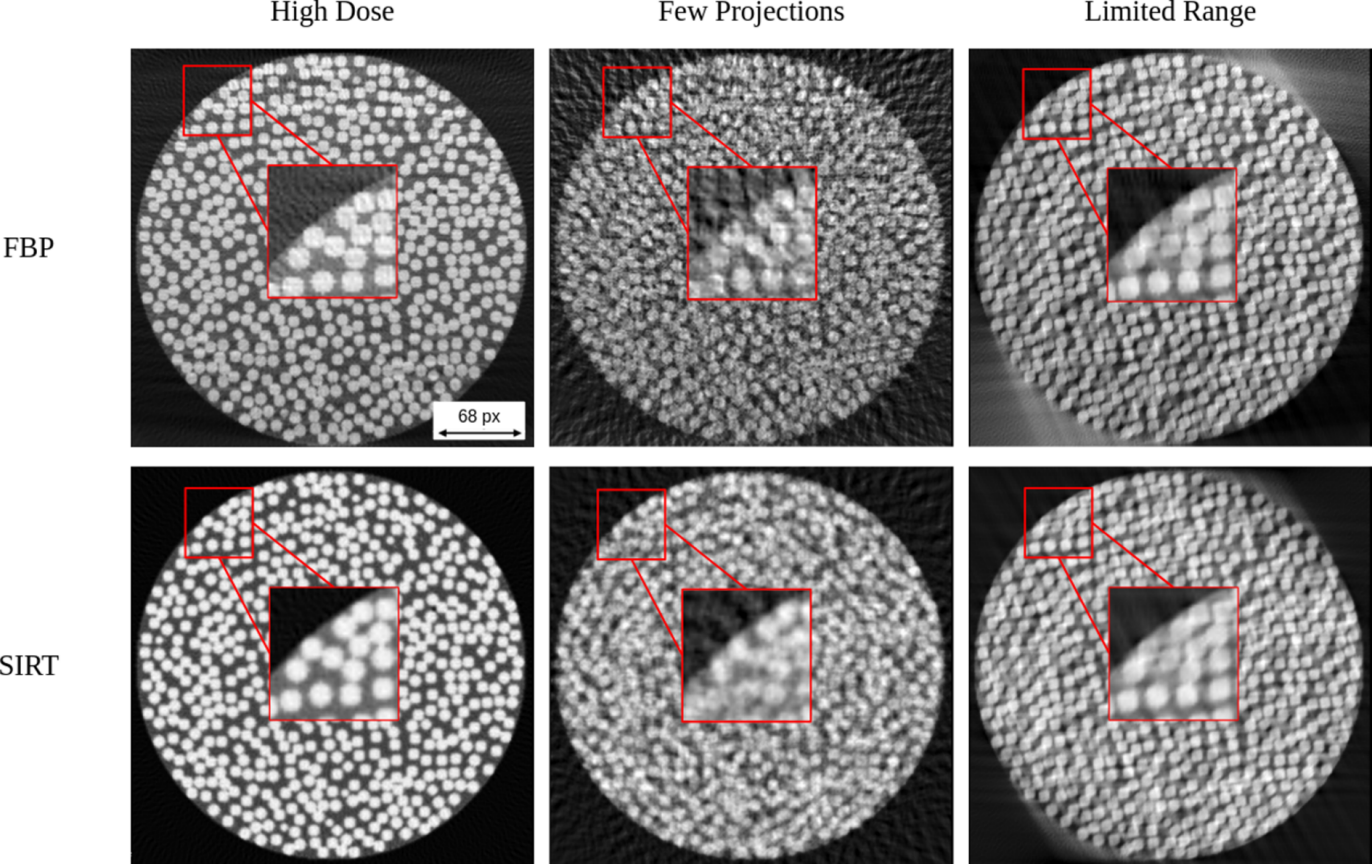
Reconstructed images for the middle axial slice of a fibre phantom, for various simulated experimental conditions [55 × 10^3^ dose (*I*_0_), 50 projections, 120° limited angular range, 49% FVF]. Given are the results for filtered back projection (FBP) and SIRT. The boxed region indicates the area shown at higher magnification to highlight the differences in boundary sharpness. The parameters used to generate these volumes are shown in Table 3 (in the supporting information).

In Fig. 6 ▸, we further examine the capacity of the simulator to evaluate the effect of various simulated scanning conditions on the generated phantoms. These conditions include variations in dose levels, projections and angular scanning range that may arise in real-world tomography setups.

**Figure 7:**
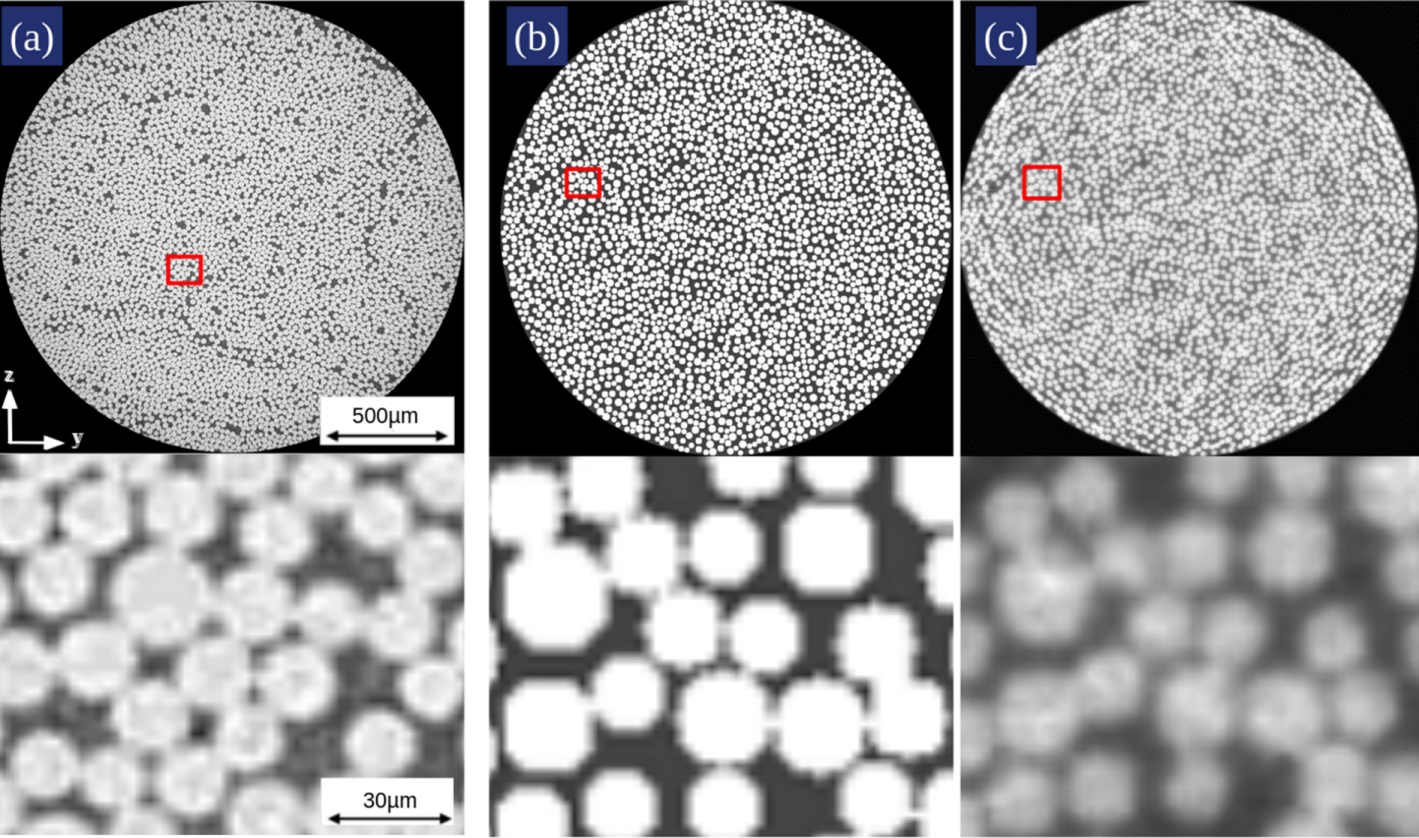
Middle axial slices (top: full view, bottom: magnified region): (*a*) real-world X-ray CT scan of a carbon FRP (Salling *et al.*, 2022 ▸), (*b*) phantom without noise and (*c*) phantom with Poisson noise. The magnified regions were selected to illustrate representative noise characteristics and local fibre diameter variation. The parameters used to generate this volume are shown in Table 4 (in the supporting information).

An example of an X-ray CT image of a unidirectional fibre-reinforced thermoset resin composite, acquired using a Zeiss Xradia Versa 520 scanner and a fibre phantom volume, is shown in Fig. 7 ▸. The scan comprised 4501 projections using a binning factor of 2, resulting in a 3D volume of 1000^3^ voxels, each with a voxel size of 1.989 µm. These cross-sectional views of the *y*–*z* plane show similarities in features between the fibre phantom, scanned and reconstructed with Poisson noise, and the real-world fibre data.

To further strengthen the validation against experimental data, we performed a quantitative comparison between the real-world CT slice and the simulated phantom slice. An initial comparison revealed minor discrepancies in the fibre diameter distribution and variability. Based on this analysis, the phantom generation parameters (*e.g.* fibre radius range and packing characteristics) were refined to better match the experimental statistics.

**Figure 8:**
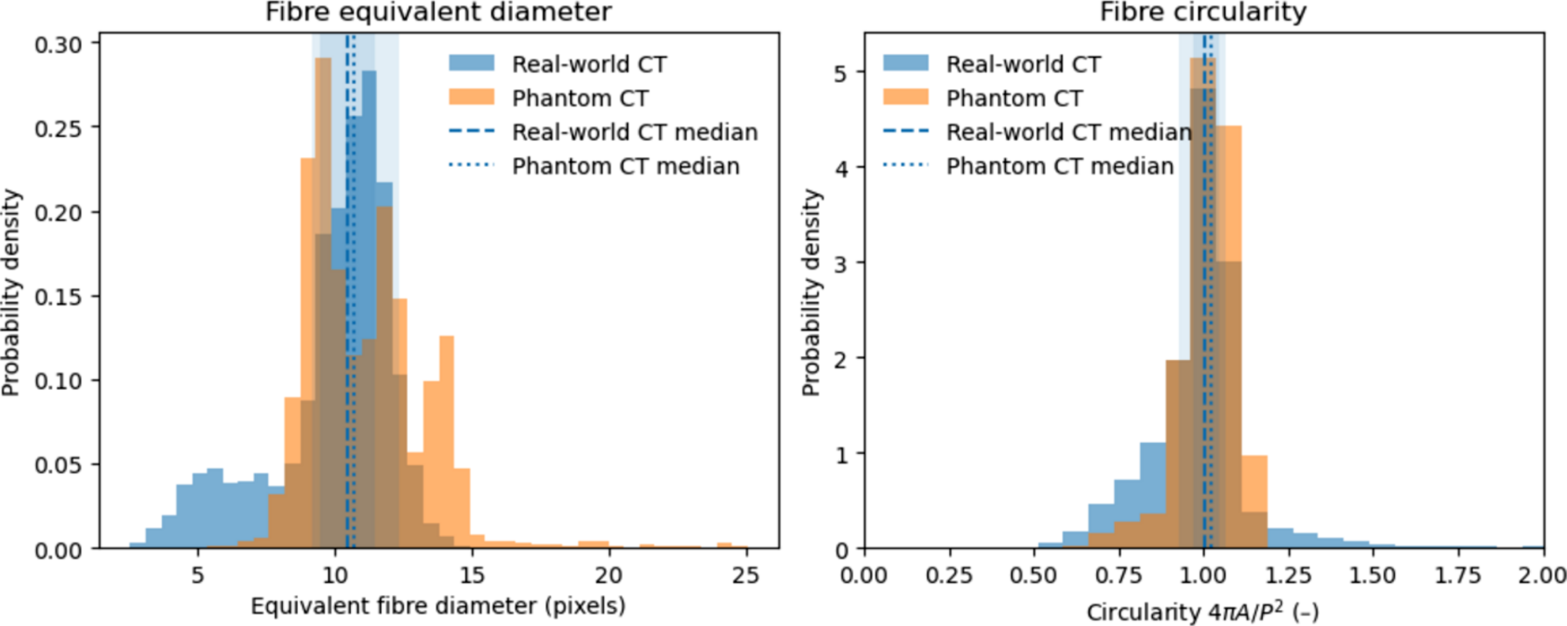
Quantitative comparison of fibre morphology between real-world CT and phantom CT cross sections. Left: probability density distributions of equivalent fibre diameter. Right: fibre circularity, defined as 4π*A*/*P*^2^, where *A* and *P* denote the area and perimeter of the segmented fibre cross sections, respectively. Circularity values close to the value of 1 indicate near-circular fibre geometries. Dashed vertical lines indicate median values for each distribution.

Shown in Fig. 8 ▸ is a quantitative 2D comparison that was performed between the real-world CT and phantom CT shown in Fig. 7 ▸. The median equivalent fibre diameter in the real-world CT is 10.46 pixels, compared with 10.52 pixels in the phantom CT, indicating the phantom reproduces the correct fibre scale while exhibiting a narrower distribution due to reconstruction blur shown in the zoomed photo of Fig. 7 ▸(*c*). The roundness of the fibres is close to the value of 1 in both cases, with median values of 1.00 for the real-world CT and 1.12 for the phantom CT, where a value of 1 corresponds to an ideal circular fibre cross section. The quantitative agreement shows that the phantom CT slice captures key features of the real-world CT fibre morphology, while observed deviations point to potential improvements in representing experimental variability, such as irregular fibre packing.

#### 3.1.1. Computational performance

The generation time strongly depends on the volume size and the number of fibres. The phantom shown in Fig. 3 ▸, with a volume size of 800 × 800 × 800 voxels and 6000 fibres, required approximately 3 h to generate. In contrast, the volume used in Fig. 5 ▸ (256 × 256 × 256 voxels, with 300, 400 and 500 fibres in the respective subfigures) required approximately 38 s, 46 s and 56 s, respectively, while the volume used in Fig. 6 ▸ required approximately 50–60 s. These results illustrate the rapid increase in computational cost for large volumes with high fibre counts, while typical volumes used for reconstruction studies can be generated within under 1 min.

### 3.2. Varying experimental scan conditions

#### 3.2.1. Fixed fibre characteristics, varying imaging conditions

**Figure 9:**
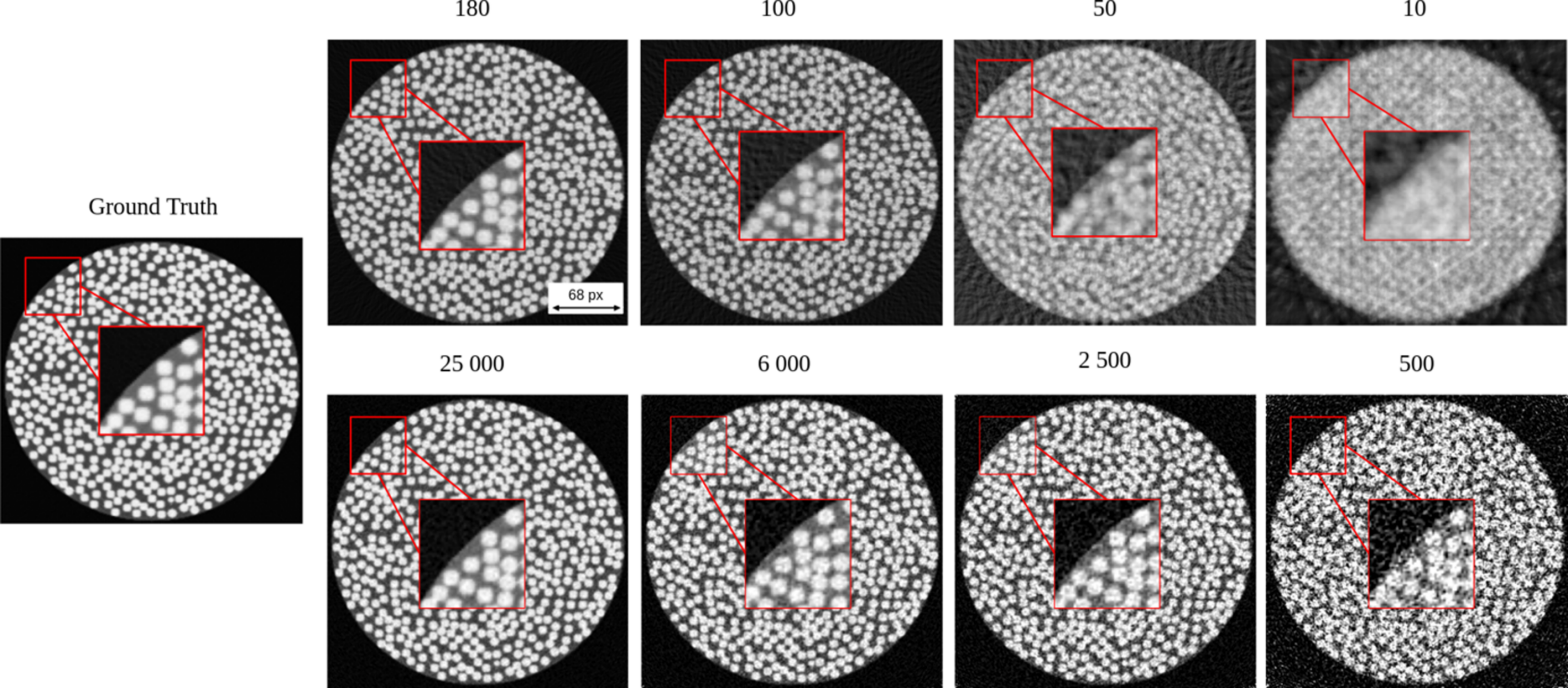
Comparison of reconstructed images as a function of the number of projections (top row) and noise levels (bottom row) against the ground truth. The boxed region indicates the area shown at higher magnification to highlight the differences in boundary sharpness. The parameters used to generate this volume are summarized in Table 4 (in the supporting information).

To further explore the capabilities of the simulator, we conducted multiple experiments under varying conditions, as shown in Fig. 9 ▸. These are axial slice reconstruction images of a [256 × 256 × 256] fibre phantom under various simulated experimental conditions. These reconstructions were acquired using a parallel beam and reconstructed using the SIRT algorithm with 200 iterations. The top row presents results for a constant noise level while varying the number of projections across the 180° angular range. The bottom row shows results for a fixed number of projections while varying the noise level. In both cases, changes in image clarity and fibre visibility illustrate the sensitivity of the reconstruction process to these parameters.

We define noise level as the amount of Poisson noise applied to the simulated projection data. This parameter is controlled by the initial photon count parameter, where a lower *I*_0_ implies a higher noise level, mimicking reduced X-ray exposure conditions.

These reconstructions illustrate the simulator’s capacity to allow users to fine-tune their experiments before undertaking CT scanning. By fine-tuning the parameters in a simulated environment, users can observe their effects on image quality and make informed adjustments. This workflow ensures that the actual scanning is carried out using an optimized set of conditions tailored to the specific imaging goals.

#### 3.2.2. Obtaining average diameter and number of fibres

To further analyse the effect of the number of projections and noise level on the quality of the reconstruction, we performed fibre analysis on the middle axial slice of the volume. We computed the average diameter of fibres and counted the number of fibres present on the slice. We estimate the number of fibres and their average diameters from 2D cross-sectional images of a reconstructed volume by applying a series of image processing and segmentation steps. This workflow is inspired by *FibreTracker’s* method to detect the centres of individual fibres (Pooja *et al.*, 2024 ▸) and implemented using classical image analysis methods (van der Walt *et al.*, 2014 ▸).

To determine the average dimaeter and count of fibres in a 2D grayscale image *I*(*x*, *y*), we apply the following steps: (1) the image is normalized and smoothed using a Gaussian filter, followed by background correction to enhance contrast and stabilize the binarization. Otsu thresholding (Otsu, 1979 ▸) is then used to obtain a binary mask, and small artefacts are removed through morphological filtering to improve segmentation quality. (2) A Euclidean distance transform (van der Walt *et al.*, 2024 ▸) is computed on the cleaned binary image, and robust markers are extracted using an *h*-maxima transform to suppress false local peaks. (3) These markers guide watershed segmentation (Zhou *et al.*, 2021 ▸), enabling reliable separation of touching fibres. (4) The resulting connected regions are uniquely labelled and their equivalent diameters are computed from their areas. The average fibre diameter is then calculated across all detected fibres.

**Figure 10:**
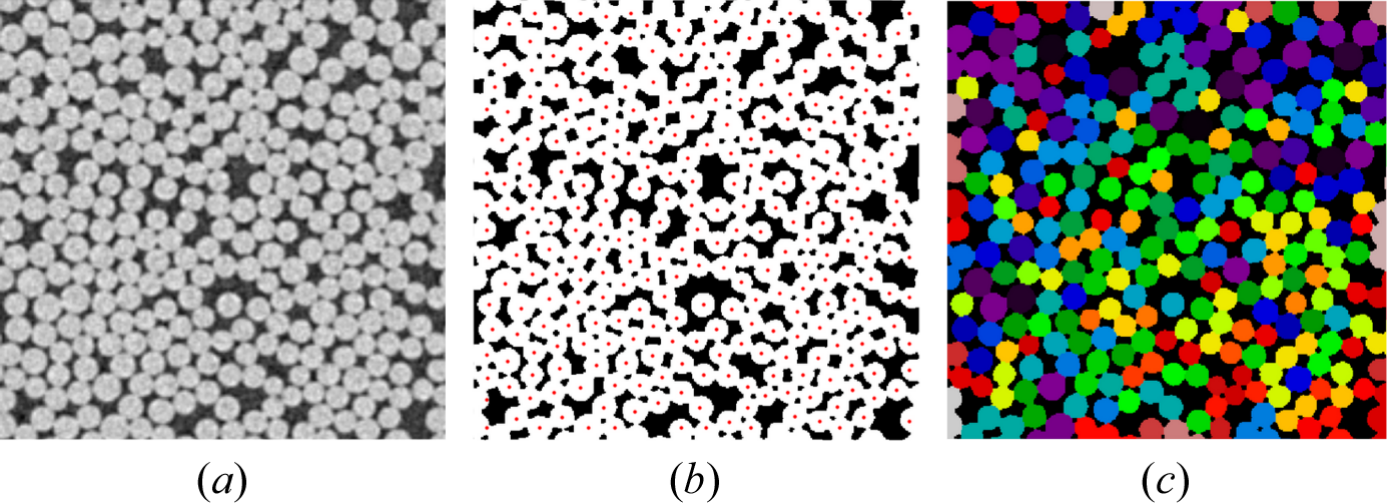
An example of applying the blob detection process: (*a*) magnified region of interest from a real-world middle slice (Salling *et al.*, 2022 ▸), (*b*) thresholded image with detected centres through identifying the peak local maximum, and (*c*) the corresponding watershed segmentation to further isolate each fibre.

By following these steps, the average fibre diameter and the number of fibres within the middle axial slice can be determined. Fig. 10 ▸ shows an example of applying this workflow to a 2D slice from a real-world CT scan.

**Figure 11:**
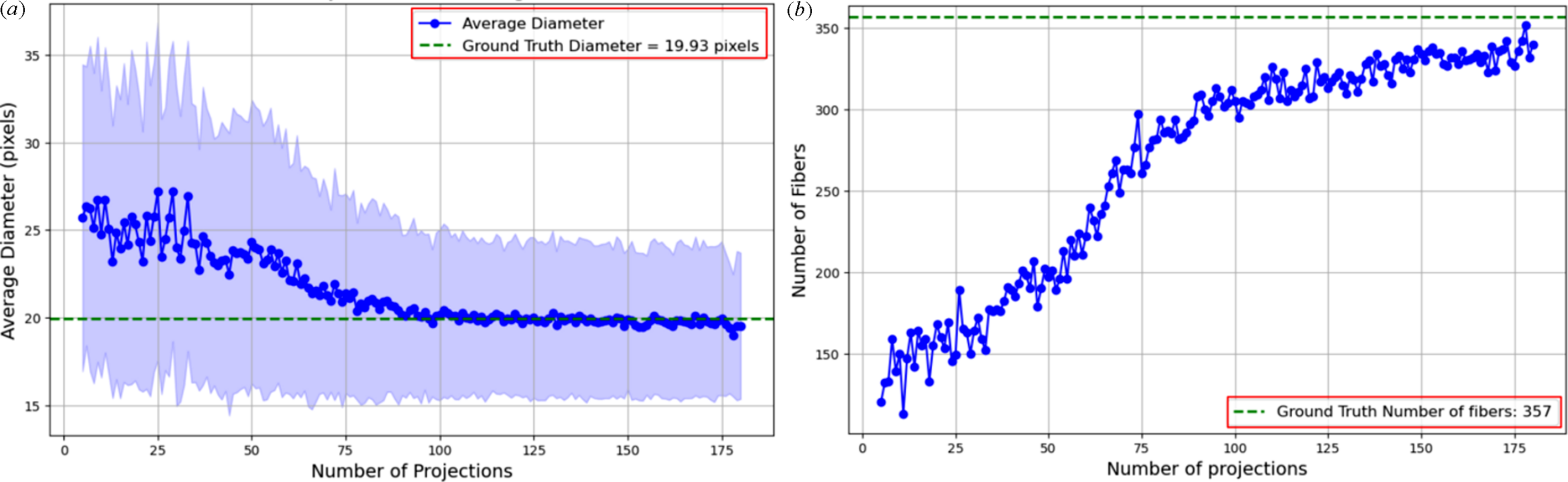
Effect of decreasing number of projections on (*a*) average fibre diameter and (*b*) number of fibres detected from the middle slice of a reconstructed volume. Each point represents measurements from a single middle slice reconstructed with a specified number of projections. The shaded region in (*a*) illustrates the variability in detected fibre diameters.

Fig. 11 ▸(*a*) illustrates the average fibre diameter in pixels as a function of the number of projections. At larger numbers, the average diameter remains consistent and close to the ground truth. However, as the number of projections decreases, the average fibre diameter increases, particularly below 75 projections. As shown in Fig. 9 ▸ (top row), we can hardly differentiate the boundaries of the fibre at 50 projections, resulting in adjacent fibres appearing fused together. This merging effect makes it difficult to distinguish individual fibres, which compromises segmentation accuracy and the reliability of diameter measurements. The larger standard deviation at the smaller range reflects an increase in uncertainty and variability. This trend is further supported by Fig. 11 ▸(*b*), which shows a decline in the number of detected fibres as boundaries merge, causing fibres to appear as single structures.

**Figure 12:**
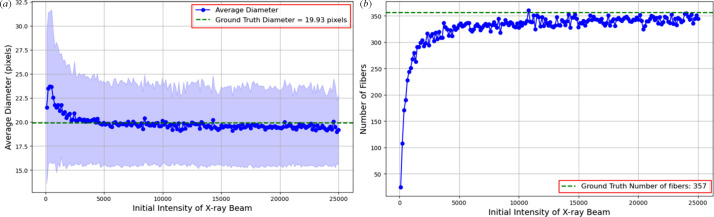
Effect of decreasing the intensity of the X-ray beam on (*a*) average fibre diameter and (*b*) number of fibres detected from the middle slice of a reconstructed volume. Each point corresponds to measurements taken from a single middle slide reconstructed with a specified X-ray intensity. The shaded region in (*a*) represents the variability in detected fibre diameters.

Fig. 12 ▸(*a*) presents the average fibre diameter in pixels as a function of the initial intensity of the X-ray beam. A decrease in the initial intensity of the X-ray beam leads to a noisier image. At higher intensities, the average diameter stabilizes close to the ground truth. However, as the initial intensity decreases, the average diameter rises. This trend indicates an increase in uncertainty and blurry effects at lower intensities, as shown in Fig. 9 ▸ (bottom row). The variability is highlighted by the larger error bars at lower intensities. The effects of varying initial intensity are further supported by Fig. 12 ▸(*b*). The graph shows how the fibre count rises and stabilizes near the ground truth as the intensity increases.

These findings suggest that reconstruction quality is highly sensitive to the number of projections and X-ray intensity. Based on our results, maintaining at least 75 projections and an initial intensity above 2.5 × 10^4^ preserves fibre boundary clarity and diameter accuracy. In our simulation, the parameter *I*_0_ represents the number of incident photons per detector bin prior to attenuation. It controls the level of Poisson noise applied to the sinogram. While *I*_0_ is a dimensionless simulation input, it serves as a proxy for real-world X-ray dose settings and can guide experimental planning for CT imaging of fibre-reinforced composites.

#### 3.2.3. Varying fibre characteristics mode, fixed imaging conditions

**Figure 13:**
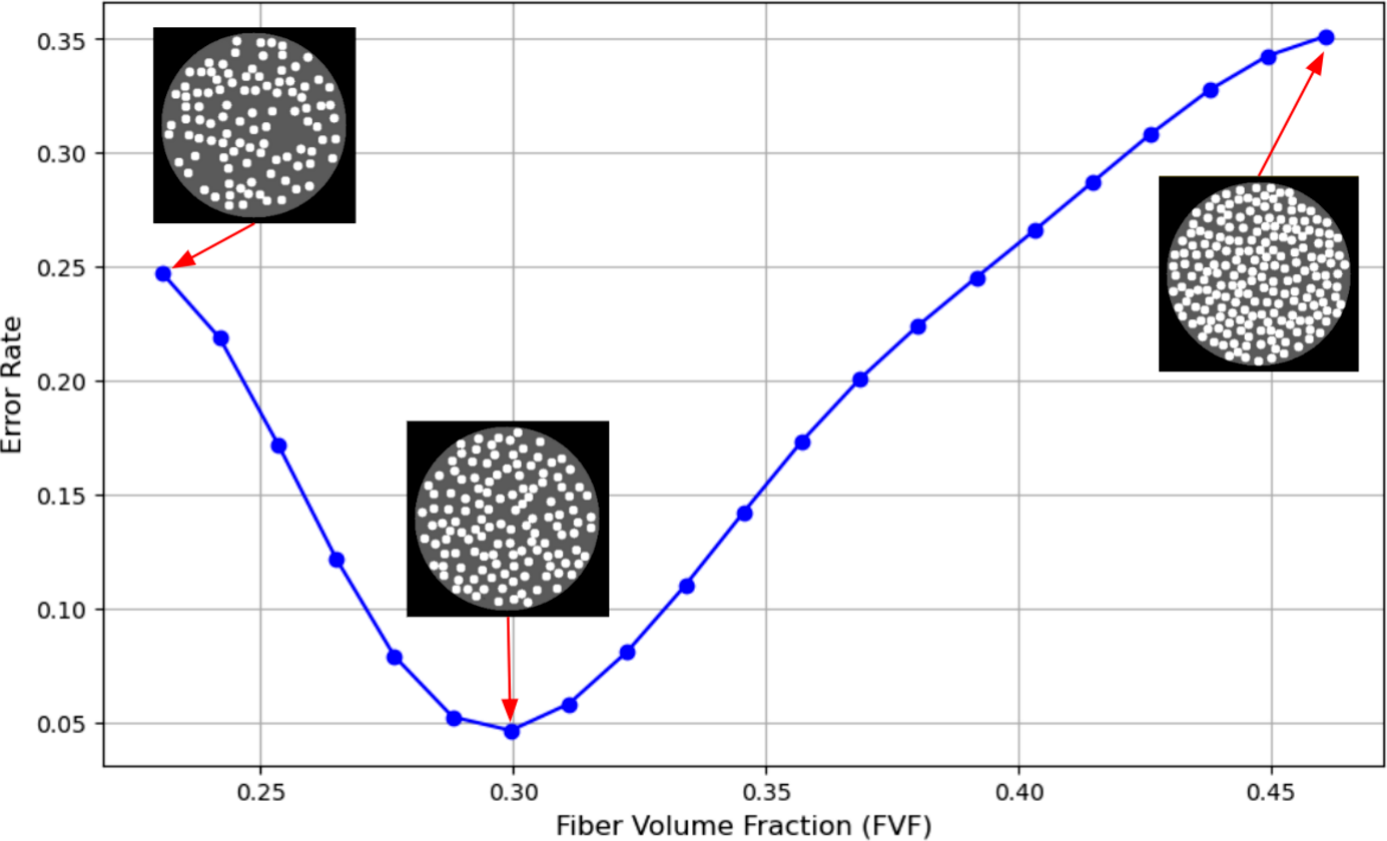
For a limited-angle scan (120°), the effect of varying FVF on the detected number of fibres is shown.

In Fig. 13 ▸, we examine the effect of increasing FVF on the detected number of fibres, based on a 120° limited-angle scan from 180 projections with a noise level of *I*_0_ = 25 × 10^3^. For each FVF value, ten phantom volumes were generated using the fibre simulator, and fibre detection was performed on the reconstructed middle slice of each volume. The average number of detected fibres across these volumes was then used to compute the error rate relative to the known ground truth.

The error rate, which quantifies the discrepancy between the detected and ground truth number of fibres, was computed as the absolute difference between the detected and ground truth counts, divided by the ground truth count.

The resulting error curve reveals two key detection regimes:

(i) Low FVF (< 0.30). With fewer fibres, each undetected fibre has a large impact on the error percentage, leading to higher variability even when fibres are visually distinct.

(ii) High FVF (> 0.30). As fibres become more densely packed, overlapping and streaking artefacts degrade separability, increasing the detection error.

Interestingly, the error rate reaches a minimum around 0.30 FVF, which corresponds to the lower bound of many practical composite materials (common 30–65% FVF) (Bhaskar & Sankaran, 2003 ▸). This finding suggests that even under limited-angle, noisy conditions, there is an optimal FVF range where detection is most reliable. For materials near this FVF, standard scanning protocols may be sufficient; however, for denser composites, enhanced imaging strategies (*e.g.* additional projections, advanced reconstruction or denoising) may be necessary to mitigate rising error.

While the plot does not encompass the full FVF range of real composites, it illustrates the existence of different regimes for fibre imaging; *FibreSimulator* enables the exploration of these for specific combinations of fibre structures and imaging parameters.

In this work, evaluation is primarily based on fibre count and average fibre diameter, which are directly relevant to common fibre analysis tasks and can be robustly estimated from reconstructed volumes. These metrics emphasize fibre detectability and geometric characterization rather than global image similarity.

Other quantitative measures could provide a complementary perspective on reconstruction quality, including segmentation-based metrics (*e.g.* intersection-over-union), centroid localization accuracy, voxel-wise similarity measures such as Structural Similarity Index (SSIM) (Wang *et al.*, 2004 ▸), with respect to the ground truth. While such metrics are not explored here, the availability of exact ground truth phantoms enables their use within the same framework when more detailed image-level or artefact-focused evaluation is required.

## 4. Conclusions

Here we introduce *FibreSimulator*, an open-source Python tool designed to generate realistic unidirectional fibre phantoms that enable fibre architectural features (*e.g.* volume fraction, fibre composition and radius, and volume dimensions) and sample geometrical features (*e.g.* holes, notches and voids) to be simulated. Furthermore, it is fully integrated with the *ASTRA* toolbox, enabling it to act as the ground truth for simulating the CT reconstruction process as a function of key CT scan parameters (*e.g.* number of projections, geometry type and reconstruction algorithm). We have demonstrated that, using the phantom generator in conjunction with the *ASTRA* toolbox enables task-specific, quantitative analysis of generated fibre structures, enabling findings such as:

(i) Reconstruction quality degrades significantly below 75 projections, leading to merged fibre boundaries, and therefore reduced segmentation accuracy.

(ii) Low simulated X-ray intensity (modelled via *I*_0_) increases Poisson noise, resulting in inflated fibre diameter estimates and inconsistent detection.

(iii) Detection error is sensitive to FVF, with a high error at both low and high FVF due to statistical sensitivity and artefact overlap, respectively.

These findings demonstrate that *FibreSimulator* can be used to explore the influence of scanning parameters on reconstruction accuracy. Future versions will support more complex fibre features such as misalignment, curvature and clustering, enabling simulation of structures observed in real materials as we see in the work of Wang *et al.* (2021 ▸). Through ongoing development, *FibreSimulator* focuses on simulating fibre morphologies and imaging artefacts, helping to make synthetic datasets more representative of real-world CT scans.

## Supplementary Material

Supporting information (Tables 3 and 4). DOI: 10.1107/S1600577526001918/gui5010sup1.pdf

## Figures and Tables

**Table 1:** List of volume generation parameters configurable in the simulator

Parameter	Description
No. of volumes	Specifies the number of volumes to be generated.
Volume dimensions	Define the size of the volume in voxels.
No. of fibres	Defines the maximum number of single fibres to be generated in the volume.
Fibre radius	Specifies the radius of individual fibres within the volume, which can be either a fixed value or randomly sampled from a specified range.
Max. and min. length	Define the range for the length of fibres.
Fibre orientation	Describes the orientation/geometric shape of the fibres. Options include straight, kinked curve, C-shaped curve, full wave and half-wave patterns. In the code, these types are specified as ‘straight’, ‘kink_curve’, ‘c_curve’, ‘full_wave_curve’ and ‘half_wave_curve’, respectively.
Defects	Refers to the types of geometric features intentionally introduced into the fibres. Options include a hole, square notch, double square notches, V-shaped notch, double V-notches, reduced-diameter section or no defect. In the code, these types are specified as ‘hole’, ‘square_notch’, ‘double_square_notch’, ‘v_notch’, ‘double_v_notch’, ‘reduced’ and ‘none’, respectively.

**Table 2:** List of tomographic simulation parameters configurable in the simulator

Parameter	Description
No. of angles	Specifies the number of projections at a 180° range used during the tomography process.
Geometry type	Indicates the X-ray beam geometry used during the simulation. Options include a parallel beam or a cone beam configuration. In the code, these types are specified as ‘parallel3d’ and ‘cone’, respectively.
Detector pixel distance	Defines the distance between the centres of two adjacent detector pixels along the horizontal and vertical axes.
Detector count	Specifies the number of detector rows and columns used in a single projection.
*I*_0_	Represents the initial intensity of the X-ray beam used in the simulation. It influences Poisson noise and interacts with the sample’s absorption – set to 0.5 in our simulation – to determine the number of photons reaching the detector (Pelt *et al.*, 2022 ▸).
Reconstruction algorithm	Specifies the algorithm used for tomographic reconstruction. Available options include iterative and analytical methods implemented on GPU: ‘SIRT3D_CUDA’ (Simultaneous Iterative Reconstruction Technique) (Gilbert, 1972 ▸) and ‘FDK_CUDA’ (Feldkamp–Davis–Kress for cone-beam geometry) (Feldkamp *et al.*, 1984 ▸).
Source-to-origin distance	Defines the distance between the X-ray source and the origin (the centre of the volume).
Origin-to-detector distance	Defines the distance from the origin (the centre of the volume) to the detector.

## Data Availability

The data supporting the results can be replicated using *FibreSimulator*, which is available in the GitHub repository Go *et al.* (2024 ▸). The details of the parameters used are provided in the supporting information.

## Appendix A Fibre growth algorithm

The fibre growth algorithm is shown in the scheme[Chem scheme1] below:
