# Supplementary material for: Fibre phantom generation using FibreSimulator: an open-source Python tool
Source: J Synchrotron Radiat. 2026 Mar 25;33(Pt 3):870–83. doi: 10.1107/S1600577526001918 (PMC13148628; doi:10.1107/S1600577526001918)
Supplement: Supplementary file 1 [file s-33-00870-sup1.pdf]

JOURNAL OF SYNCHROTRON RADIATION

Volume 33 (2026)

Supporting information for article:

Fibre phantom generation using *FibreSimulator*: an open-source Python tool

Mary Chris Roperos Go, Daniël M. Pelt, Anirudh Kohli, Philip J. Withers and K. Joost Batenburg

Table 3: Volume and tomographic parameters for Figures 3, 5, and 6. A dash (–) indicates a parameter not specified.

**Parameter**	**Fig. 3**	**Fig. 5**	**Fig. 6**
Volume dimensions	[800, 800, 800]	[256, 256, 256]	[256, 256, 256]
Number of fibres	6000	300 / 400 / 500 (subfigures)	500
Fibre radius	[4, 6]	4/ 5 / 6	4
Max. length	750	250	250
Min. length	350	50	50
fibre curve intensity	kink curve	straight	straight
Defects	hole	none	none
Number of projections	180	180	50
Geometry type	parallel beam	parallel beam	parallel beam
Detector pixel distance (row)	1.0	1.0	1.0
Detector pixel distance (column)	1.0	1.0	1.0
Detector count (x)	1000	512	512
Detector count (y)	1000	512	512
I_0	5.5×10^5	5.5×10^5	5.5×10^4
Reconstruction algorithm	SIRT	SIRT	FBP / SIRT
Source-to-origin distance	3200	1000	1000
Origin-to-detector distance	1600	500	500

Table 4: Volume and tomographic parameters for Figures 7 and 9.

**Parameter**	**Fig. 7**	**Fig. 9**
Volume dimensions	[800, 800, 800]	[256, 256, 256]
Number of fibres	7000	1000
fibre radius	[5, 7]	4
Max. length	700	200
Min. length	100	80
fibre curve intensity	straight	straight
Defects	none	none
Number of projections	180	180
Geometry type	cone beam	parallel beam
Detector pixel distance (row)	1.0	1.0
Detector pixel distance (column)	1.0	1.0
Detector count (x)	1024	512
Detector count (y)	1024	512
I_0	5×10^6	5.5×10^5
Reconstruction algorithm	FDK	SIRT
Source-to-origin distance	1600	500
Origin-to-detector distance	800	500
